# Characterisation of Colistin -Resistant Enterobacterales and *Acinetobacter* Strains Carrying *mcr* Genes from Asian Aquaculture Products

**DOI:** 10.3390/antibiotics10070838

**Published:** 2021-07-09

**Authors:** Alžběta Kalová, Tereza Gelbíčová, Søren Overballe-Petersen, Eva Litrup, Renáta Karpíšková

**Affiliations:** 1 Department of Microbiology and Antimicrobial Resistance, Veterinary Research Institute, 621 00 Brno, Czech Republic; gelbicova@vri.cz (T.G.); karpiskova@vri.cz (R.K.); 2 Department of Experimental Biology, Faculty of Science, Masaryk University, 602 00 Brno, Czech Republic; 3 Statens Serum Institut, 2300 Copenhagen, Denmark; SOVP@ssi.dk (S.O.-P.); EVL@ssi.dk (E.L.)

**Keywords:** colistin, resistance, *mcr* genes, plasmids

## Abstract

Aquaculture systems are widely recognised as hotspots for horizontal gene transfer, and the need for screening for bacteria carrying antimicrobial resistance genes in aquaculture systems is becoming more important. In this study, we characterised seventeen bacterial strains (*Escherichia coli*, *Klebsiella pneumoniae*, *Acinetobacter baumannii,* and *A. nosocomialis*) resistant to colistin originating from retailed aquaculture products imported from Vietnam to the Czech Republic. The *mcr-1.1* gene was found located on plasmid types IncHI2, IncI2, and IncX4, as well as on the rarely described plasmid types IncFIB-FIC and IncFIB(K), phage-like plasmid p0111, and on the chromosome of *E. coli*. One *E. coli* strain carried the *mcr-3.5* gene on IncFII(pCoo) plasmid in addition to the *mcr-1.1* gene located on IncHI2 plasmid. *K. pneumoniae* was found to carry the *mcr-1.1* and *mcr-8.2* genes on IncFIA(HI1) plasmid. The *mcr-4.3* gene was found on similar untypeable plasmids of *A. baumannii* and *A. nosocomialis* strains, pointing to the possible interspecies transfer of plasmids carrying the *mcr-4* gene. Our results highlight that some aquaculture products of Asian origin can represent an important source of variable plasmids carrying *mcr* genes. The results showed an involvement of phages in the incorporation of the *mcr-1* gene into plasmids or the chromosome in *E. coli* strains from aquaculture. The detection of *E. coli* with the *mcr-1* gene in the chromosome points to the risks associated with the stabilisation of the *mcr* genes in the bacterial chromosome.

## 1. Introduction

Large amounts of antibiotics have been reported to be used in Asia, not only in public health but also as feed additives for the prevention or treatment of bacterial diseases in animal production, including aquaculture [1]. Antibiotic residues entering rivers and water used for aquaculture may then pose serious environmental risks to food production [2] because residues can persist there for a long time [3]. This fact is one of the reasons why antimicrobial resistance surveillance should be implemented in aquaculture farm products.

Colistin (CT) is a last-resort antibiotic used mainly for the treatment of infections caused by multidrug-resistant Gram-negative bacteria [4]. Resistance to colistin was long thought to be only chromosomally encoded, but this perspective changed in 2015 when Liu et al. described plasmid-mediated colistin resistance encoded by the *mcr-1* gene [5]. Since the first report, the *mcr-1* gene has been found in bacteria from various sources worldwide [6]. Subsequently described *mcr-2* to *mcr-10* genes [7,8,9,10,11,12,13,14,15] have emerged. The *mcr* genes have been found localised on various plasmid types as well as integrated in the chromosome [6], and the dissemination of *mcr*-mediated resistance represents a significant threat in the spreading of colistin resistance in clinically significant pathogenic bacteria—e.g., *Escherichia coli* [16], *Klebsiella pneumoniae* [17], *Salmonella enterica* [18], and *Acinetobacter baumannii* [19].

Plasmids can be characterised by many ways, and the most common is to divide them by their incompatibility (Inc) groups. Currently, there are 28 Inc groups of plasmids in *Enterobacteriaceae* [20]. The host range can be limited only to *Enterobacteriaceae* (e.g., IncF or IncX) or can be broader (e.g., IncA/C, IncH, or IncP) [20]. According to Carattoli [21], the major plasmid families associated with antimicrobial resistance genes (ARGs) in *Enterobacteriaceae* are IncF, IncA/C, IncL/M, IncI1, IncHI2, and IncN. Some of these plasmid groups can be linked to specific resistance genes—e.g., IncF plasmids are frequently described carrying genes encoding resistance to extended-spectrum beta-lactams, carbapenems, aminoglycosides, or fluoroquinolones; IncI2, IncX4, and IncP plasmids are associated with resistance to colistin encoded by the *mcr-1* gene; IncHI1 and IncHI2 plasmid are reported to be associated with multidrug resistance (including colistin resistance *mcr-1* and *mcr-3* genes); and ColE plasmids are reported to carry colistin resistance genes *mcr-4* and *mcr-5* [20]. The plasmids of *Acinetobacter baumannii* belong to a limited number of plasmid lineages and only around one third of them carry any ARGs (the most frequent are genes encoding resistance to aminoglycosides, beta-lactams, or sulphonamides) [22]. Plasmids associated with genes encoding resistance to colistin often carry the *mcr-4.3* gene [19,23,24]. The ARGs are often found located close to mobile elements such as insertion sequences (IS), which help them to spread between different plasmids and chromosomes [25]. Insertion sequences have been described as the most abundant and ubiquitous genes in nature [26], and some specific IS can be linked to particular ARG—e.g., IS*Apl1* is associated with the *mcr-1* gene [5].

Although bacteria carrying *mcr* genes in poultry, pork, or beef meat have been described extensively [6,27], little is known about the detailed characteristics of plasmids carrying colistin resistance genes from aquaculture products [28].

In China, several *mcr-1*-positive bacterial strains have been reported in aquaculture products: *E. coli* from grass carp carrying the *mcr-1* gene on IncI2, IncP, and IncX4 plasmids or in the chromosome [29]; *E. coli* and *K. pneumoniae* from duck-fish integrated fisheries, slaughterhouses, and fish markets with *mcr-1* on IncHI2, IncI2, IncX4, and IncP plasmids [30]; and a first detected *Vibrio parahaemolyticus* bearing the *mcr-1* gene on a transferable IncX4 plasmid originating from shrimps [31]. In Vietnam, extended-spectrum beta-lactamase (ESBL) producing *E. coli* harbouring the *mcr-1* gene isolated from fish gut was detected by PCR in the Mekong delta [32].

In Europe, *mcr-1*-positive *E. coli* has been found in pangasius fillets and prawns imported from Vietnam to Denmark [33]. Similarly, in Norway, a scampi imported from Bangladesh was found to be positive for *E. coli* carrying the *mcr-1* gene on the IncHI2 plasmid type [34].

The detection of the *mcr-3* gene in aquaculture has been reported mostly as *mcr-3*-like gene in the bacterial species *Aeromonas* isolated from fish [35,36]. These aquatic bacteria are believed to be “the source” of the *mcr* genes and their phosphoethanolamine transferases show a significant identity with the *mcr-3* gene found in *E. coli* [37]. None of the rest of the currently described *mcr* genes have been detected in bacterial isolates from aquaculture products as of yet.

This study aims to provide a detailed characterisation of *mcr*-positive strains isolated from retailed aquaculture products imported from Vietnam to the Czech Republic, with a special emphasis on the localisation of *mcr* genes along with genes encoding resistance to other antimicrobials.

## 2. Results

### 2.1. Bacterial Isolates

Seventeen bacterial isolates resistant to colistin were acquired from aquaculture products (frog legs, crab meat, and pangasius meat) originating from Vietnam and retailed in the Czech Republic in 2019. The tested collection consisted of fourteen *E. coli* isolates, one *K. pneumoniae*, one *A. baumannii,* and one *A. nosocomialis* (Table 1).

### 2.2. Colistin Susceptibility

All the tested isolates were resistant to colistin, with minimum inhibitory concentrations (MICs) > 2 mg/L (Table 1). The MIC of *E. coli* strains ranged from 4 to 8 mg/L, and only one *E. coli* strain CT226 carrying two copies of the *mcr-1* gene had an MIC > 16 mg/L. On the other hand, another *E. coli* strain CT262 with both *mcr-1* and *mcr-3* genes had an MIC = 4 mg/L. The MIC of *K. pneumoniae* CT251 with *mcr-1* and *mcr-8* genes and *Acinetobacter* spp. strains CT237 and CT263 carrying the *mcr-4* gene was >16 mg/L.

### 2.3. Multi-Locus Sequence Typing (MLST)

Whole-genome sequencing was applied and the 7 locus MLST showed a high variability between strains of *E. coli*. Only ST48 was identified in more than one strain (n = 4) originating from two samples of frog legs. Nevertheless, the strains varied in terms of their contents of ARGs and plasmids (Table S1). *A. nosocomialis* belonged to ST279, *A. baumannii* to ST490, and ST11 to the *K. pneumoniae* strain (Table 1).

### 2.4. Detected mcr Genes

The sequences of all strains were checked for the presence of the *mcr* genes. All tested *E. coli* (4 strains from one meat sample of pangasius fish, 2 strains from one meat sample of blue swimmer crab, and 8 strains from two samples of frog legs) and *K. pneumoniae* (1 strain from the sample of frog legs) carried the *mcr-1.1* gene. In contrast to Enterobacterales strains, *A. baumannii* from the sample of frog legs and *A. nosocomialis* from the meat sample of pangasius fish carried the *mcr-4.3* gene. One *E. coli* strain (CT262) originating from frog legs carried the *mcr-3.5* gene in addition to *mcr-1.1.* The only *K. pneumoniae* strain tested carried *mcr-8.2* together with *mcr-1.1* (Table 1).

### 2.5. Genetic Environment of the mcr Genes on Plasmids

**Figure 1:**
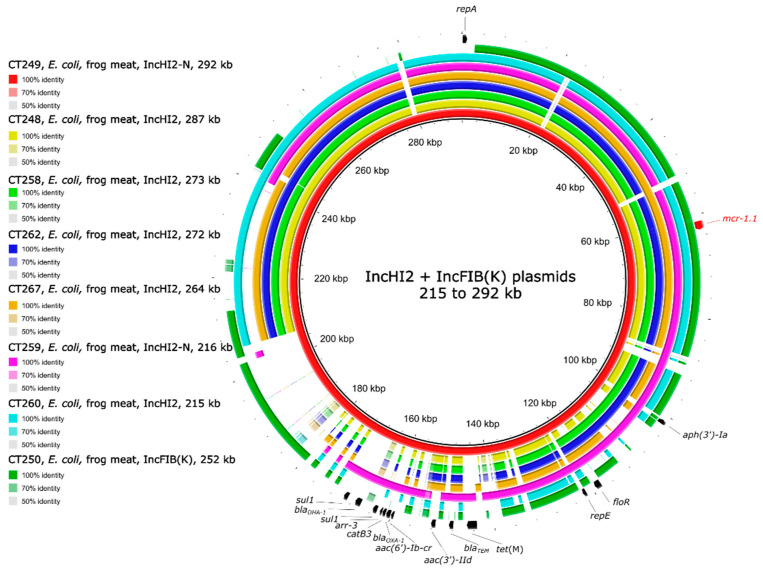
Genetic comparison of IncHI2 plasmids of *E. coli* strains CT249, CT248, CT258, CT262, CT267, CT259, CT260, and IncFIB(K) plasmids of *E. coli* strain CT250. The identity was calculated in comparison to plasmids of strain CT249 (red circle). The outer arrows show the ARGs, insertion sequences, and/or replication proteins present in the reference (red) plasmid.

**Figure 2:**
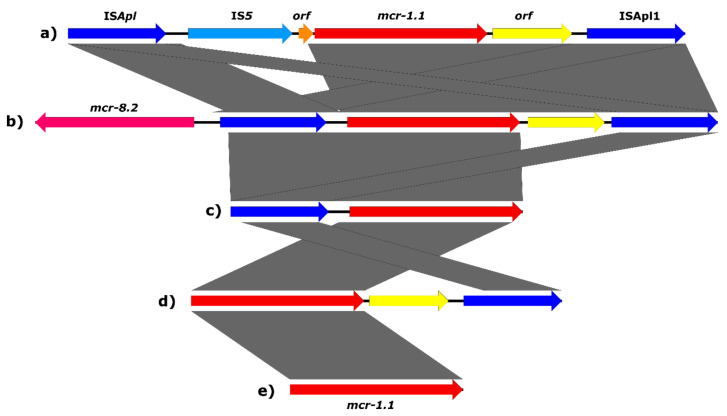
Types of genetic surroundings around the *mcr-1.1* gene in tested strains of Enterobacterales. (**a**) The *mcr-1.1* gene in the chromosome of *E. coli* CT230 in an atypical Tn*6330* with IS*5* and open reading frame (*orf*) inserted upstream of the *mcr-1.1* gene. (**b**) The *mcr-1.1* gene in the complete Tn*6330* with *mcr-8.2* located upstream of the transposon in *K. pneumoniae* CT251 plasmid. (**c**) IS*Apl1* upstream of the *mcr-1.1* gene on plasmids in *E. coli* strains CT225, CT228, CT229, CT248, CT250, CT258, CT260, CT262, and CT267. (**d**) *orf* and IS*Apl1* downstream of the *mcr-1.1* gene in *E. coli* CT227 (chromosome). (**e**) No ISApl1 sequence on plasmids in *E. coli* strains CT226, CT249, and CT259.

To determine the localisation of the *mcr* genes on plasmids or in the chromosome, long-read sequencing was performed. The IncHI2 plasmid type with the *mcr-1.1* gene was the most prevalent (n = 7) and was present in *E. coli* originating from frog legs (Table 1). The IncHI2 plasmids were approx. 215 to 292 kb in size and carried the *mcr-1.1* gene in addition to multiple other ARGs (Figure 1). Plasmids of *E. coli* strains CT249 and CT259 carried a replicon type IncN in addition to IncHI2. The IS*Apl1* transposase associated with the *mcr-1* gene was found upstream of the *mcr-1* gene in five out of seven of the IncHI2 plasmids (Figure 2c). The *mcr-1.1* gene in the *E. coli* strain CT250 originating from frog legs was found on IncFIB(K) plasmid together with other ARGs (Table S1). A plasmid type IncFIB(K) of *E. coli* strain CT250 shared around 50% coverage with the IncHI2 plasmid type (Figure 1). The main shared sequence included the *mcr-1.1* gene. The *mcr-1.1* gene in CT250 had one single-nucleotide polymorphism (SNP) in comparison with the reference gene *mcr-1.1*, but it did not lead to a change in amino acid. The IS*Apl1* transposase was found upstream of the *mcr-1.1* gene in CT250 (Figure 2c).

**Figure 3:**
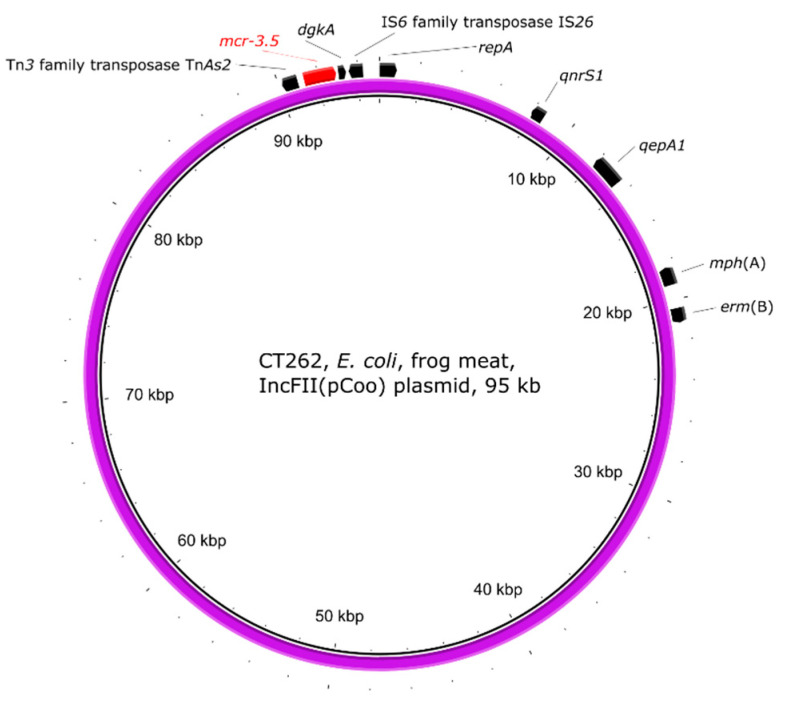
Visualisation of the *mcr-3.5*-carrying IncFII(pCoo) plasmid of *E. coli* strain CT262. The outer arrows show the ARGs, insertion sequences, and other genes and/or replication proteins present in the plasmid.

The *E. coli* strain CT262 with *mcr-1.1* on IncHI2 plasmid also carried the *mcr-3.5* gene found on IncFII(pCoo) plasmid (Figure 3) containing other ARGs. Tn*3* family transposase Tn*As2* associated with the *mcr-3* gene was found upstream, while *dgkA* diacylglycerol kinase and IS*6* family transposase IS*26* were found downstream of the *mcr-3.5* gene.

**Figure 4:**
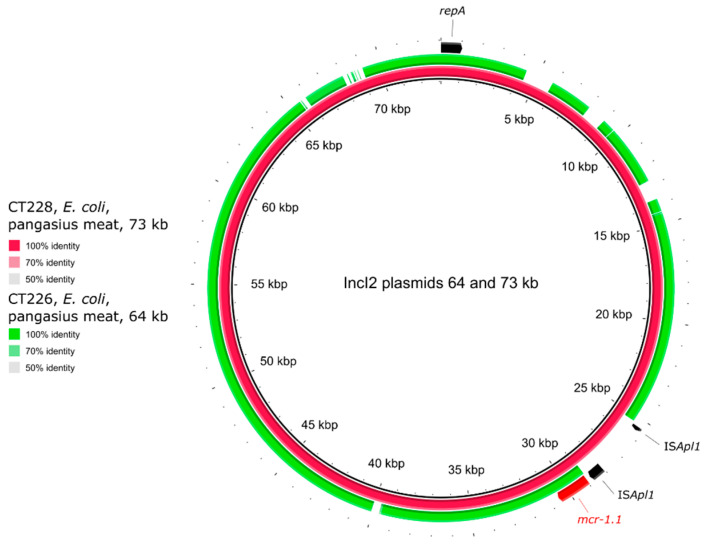
Genetic comparison of the IncI2 plasmids of *E. coli* strains CT228 and CT226. The identity was calculated in comparison to the plasmids of strain CT228 (red circle). The outer arrows show the ARGs, insertion sequences, and/or replication proteins present in the reference (red) plasmid.

**Figure 5:**
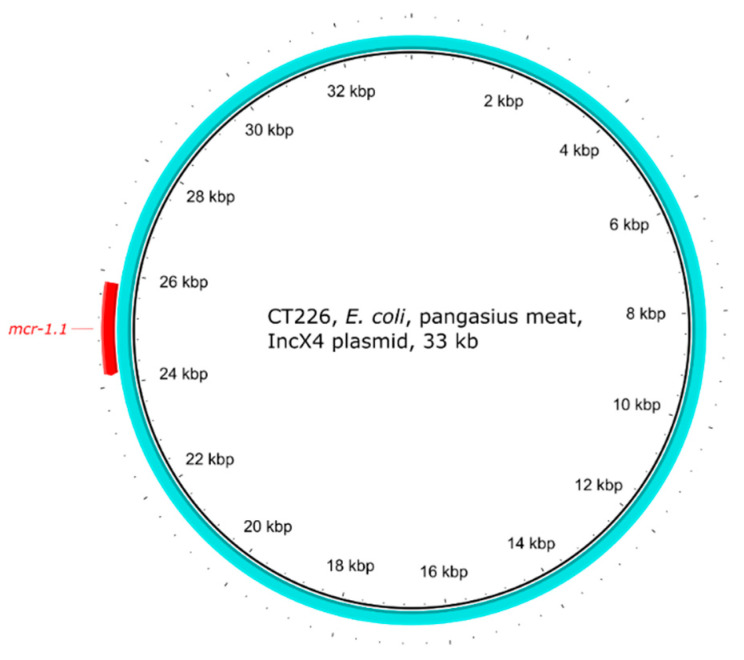
Visualisation of the *mcr-1.1*-carrying IncX4 plasmid of *E. coli* strain CT226. The outer arrow shows the ARG present in the plasmid.

The IncI2 plasmid type (n = 2) was found in *E. coli* from pangasius (Table 1). The IncI2 plasmids were approx. 64 and 73 kb in size and carried only *mcr-1.1* as ARG (Figure 4). The IS*Apl1* transposase was found twice (once truncated) upstream of the *mcr-1.1* gene located on IncI2 plasmid in strain CT228. No IS*Apl1* was found on the same plasmid type in strain CT226 (Figure 2e). The strain CT226 carried a second copy of the *mcr-1.1* gene on a plasmid of IncX4 type, size approx. 33 kb, with no other ARGs (Figure 5).

**Figure 6:**
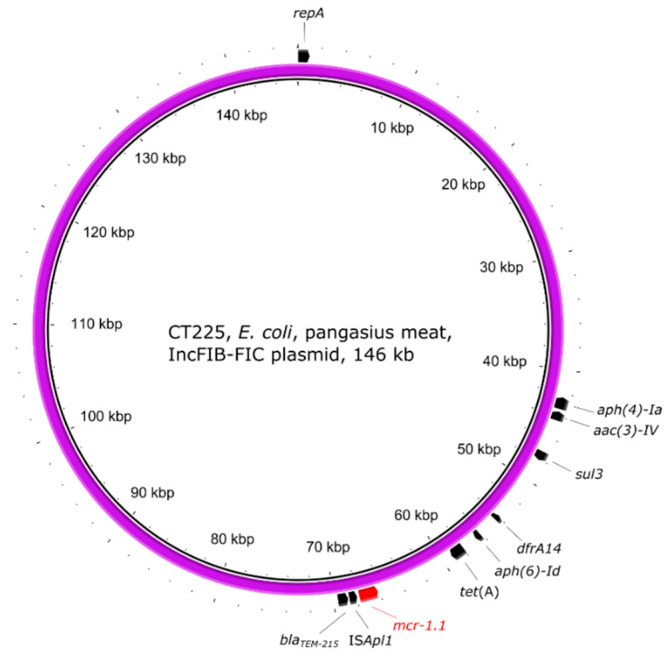
Visualisation of the *mcr-1.1*-carrying IncFIB-FIC plasmid of *E. coli* strain CT225. The outer arrows show the ARGs, insertion sequences, and/or replication proteins present in the plasmid.

In the *E. coli* strain CT225, originating from pangasius, the *mcr-1.1* gene was localised on a IncFIB(AP001918)-FIC(FII) plasmid carrying several other resistance genes (Figure 6). The IS*Apl1* transposase was found upstream of the *mcr-1.1* gene (Figure 2c).

**Figure 7:**
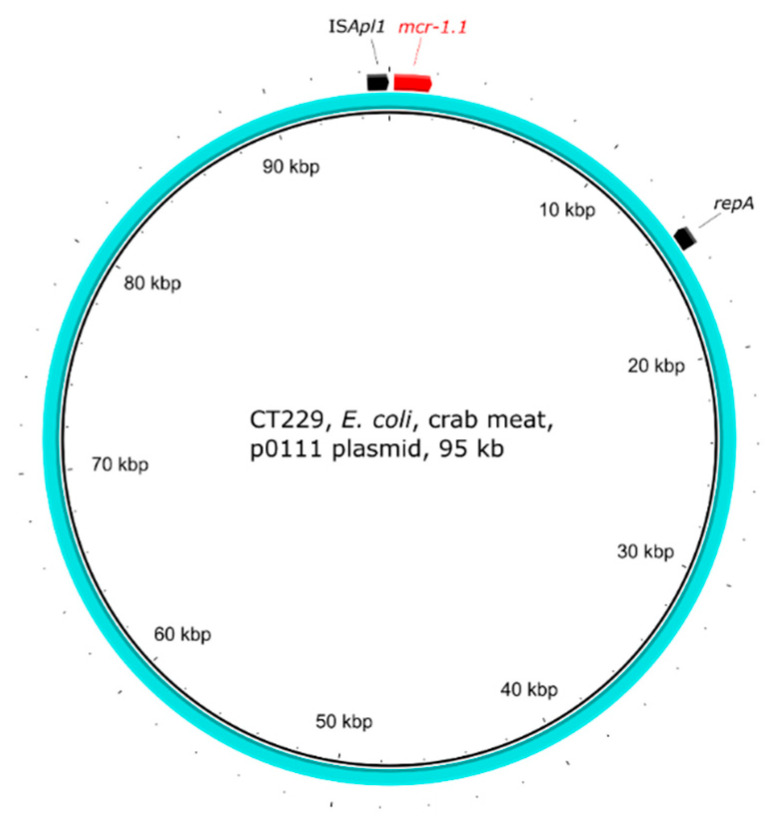
Visualisation of the *mcr-1.1*-carrying p0111 phage-like plasmid of *E. coli* strain CT229. The outer arrows show the ARGs, insertion sequences, and/or replication proteins present in the plasmid.

The *E. coli* strain CT229 originating from crab meat carried the *mcr-1.1* gene on a p0111 plasmid type (Figure 7). When annotating the plasmid, many phage related proteins were found. Therefore, the plasmid sequence was analysed by Phaster [38] and a P1 phage was found in 98% of the plasmid sequence. The P1 phage was found to be intact, with a score of >90. The BLASTn results showed that it was 98% identical to phage P1 (accession number AF234172) at a 77% coverage.

**Figure 8:**
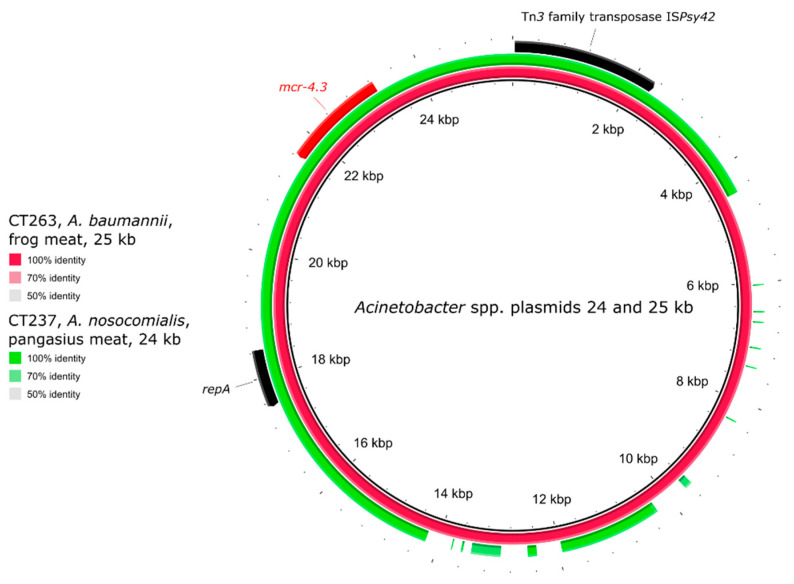
Genetic comparison of Acinetobacter spp. plasmids of strains CT263 and CT237. The identity was calculated in comparison to the plasmids of strain CT263 (red circle). The outer arrows show the ARGs, insertion sequences, and/or replication proteins present in the reference (red) plasmid.

The plasmids of *Acinetobacter* spp. were not typed using PlasmidFinder [39], since the database focuses mainly on *Enterobacteriaceae* members and Gram-positive plasmid typing. The two *Acinetobacter* plasmids carried only the *mcr-4.3* gene as ARG (Figure 8). The comparison showed a high identity in an approx. 17 kb segment of the plasmids of approx. 24 and 25 kb sizes. Tn*3* family transposase IS*Psy42* was found upstream of *mcr-4.3* in both plasmids.

**Figure 9:**
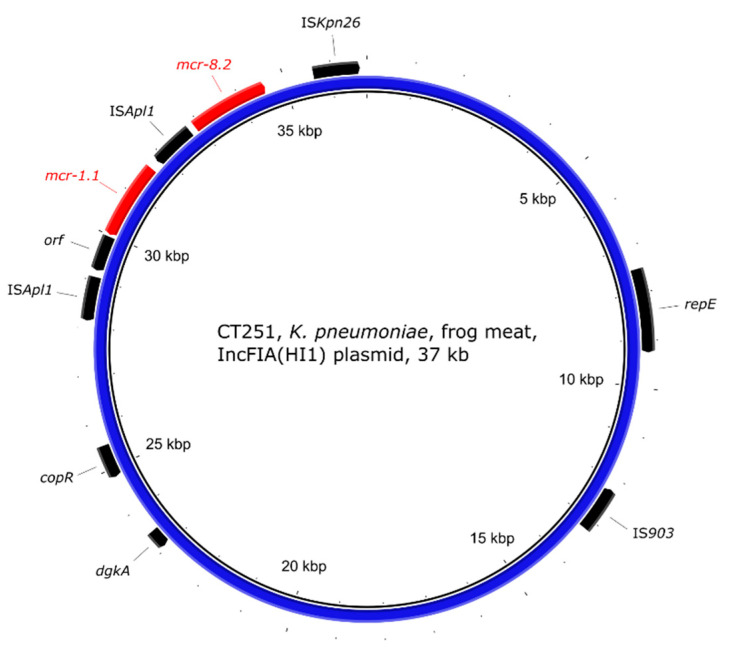
Visualisation of the *mcr-1.1* and *mcr-8.2* carrying IncFIA(HI1) plasmid of *K. pneumoniae* strain CT251. The outer arrows show the ARGs, insertion sequences, other genes, and/or replication proteins present in the plasmid.

The *K. pneumoniae* strain CT251 originating from frog legs carried both *mcr-1.1* and *mcr-8.2* on a IncFIA(HI1) plasmid of size approx. 37 kb (Figure 9). No other ARGs were found to be located on the plasmid. The transposon Tn*6330* (IS*Apl1*-*mcr-1.1*-*orf*-IS*Apl1*) was found around the *mcr-1.1* gene and the *mcr-8.2* gene was found upstream of Tn*6330* (Figure 2b).

### 2.6. Genetic Surroundings of the mcr-1 Gene in the Chromosome

The *mcr-1.1* gene was found in the chromosome in two *E. coli* strains, CT227 and CT230, originating from meat samples of pangasius and crab. Both strains carried several other ARGs in their chromosomes (Table S1). When examining the genetic context of the *mcr-1.1* gene in CT227, IS*Apl1* transposase was found downstream, along with several phage-related sequences around the *mcr-1.1* gene (Figure 2d). After submitting the sequence to Phaster [38], the results showed an Enterobacteria lambda phage (NC_001416) present around the *mcr-1.1* gene with a questionable score of 70–90. BLASTn results showed a 98% identity at a 64% coverage with the phage sequence. On the contrary, no phage sequences were found around the *mcr-1.1* gene in the CT230 strain. The context of the *mcr-1.1* gene in the CT230 strain was IS*Apl1*-IS*5*-*orf*-*mcr-1.1*-*orf*-IS*Apl1* (Figure 2a), and multiple copies of IS*Apl1* were present throughout the whole chromosome.

### 2.7. Co-Occurrence of Genes Encoding Resistance to Different Classes of Antimicrobials

The strains of *E. coli* were generally multiresistant, carrying genes encoding resistance to at least six antibiotic classes found by ResFinder [40]. All *E. coli* strains (n = 8) originating from frog legs carried genes encoding resistance to rifampicin and were also carrying genes encoding ESBL (*bla*_CTX-M-55_, *bla*_OXA-1_ or *bla*_VEB_) (Table S1). No genes encoding resistance to carbapenems were found. Genes encoding resistance to fluoroquinolones (*qnrS1*, *aac(6’)-Ib-cr,* or *qepA1*) were found in eleven strains of *E. coli* (Table S1).

*K. pneumoniae* strain CT251 with both *mcr-1.1* and *mcr-8.2* genes carried both the *bla*_CTX-M-65_ and *bla*_SHV-182_ genes encoding ESBL. Both *Acinetobacter* spp. strains carried the *mcr-4.3* gene. *A. nosocomialis* strain CT237 carried *bla*_ADC-68_ gene encoding ESBL. On the other hand, *A. baumannii* strain CT263 carried *bla*_ADC-25_, encoding a cephalosporinase.

The complete resistance genes profiles with their localisation on the plasmid or chromosome of all tested bacterial strains are presented in Table S1.

## 3. Discussion

In this study, all tested isolates of Enterobacterales and *Acinetobacter* spp. originating from retailed aquaculture products with resistance to colistin were found to be positive for the presence of different variants of the *mcr* genes.

In this study, the MIC of *E. coli* strain CT226 with two copies of the *mcr-1* gene (on IncX4 and IncI2 plasmids) was 16 mg/L. Interestingly, *E. coli* strain CT262 with two copies of the *mcr* genes (*mcr-1* and *mcr-3*) had an MIC = 4 mg/L. The occurrence of multiple copies of *mcr* genes in one strain does not have to lead to increased resistance to colistin—e.g., in the case of the co-occurrence of *mcr-1* and *mcr-3* in *E. coli* from cattle in Spain (MIC = 4 mg/L) [41] or the co-occurrence of the *mcr-1* gene on plasmid and in the chromosome of *E. coli* from swine in China (MIC = 4 mg/L) [42]. *E. coli* was predominantly associated with the *mcr-1* gene, which is consistent with the worldwide prevalence of the *mcr-1* gene in Enterobacterales of different origin [6]. In a study on retailed meat (poultry, beef, pork, and rabbit) from the Czech Republic, the *mcr-1* gene was also found to be predominant in *E. coli* strains [43]. The MLST of *E. coli* varied and no correlation was observed.

The co-occurrence of *mcr-1* and *mcr-8* genes on one plasmid was observed in *K. pneumoniae* strain CT251 in this study. The co-occurrence of *mcr-1* and *mcr-8* genes in *K. pneumoniae* has been described before, but the genes were located on two different plasmids [44].

Colistin resistance in *Acinetobacter* species was long thought to be only chromosomally encoded [45]; however, recently several studies have reported the occurrence of plasmid mediated colistin resistance in *Acinetobacter* spp. The *mcr-1* gene in *Acinetobacter* species has been found in clinical strains from China [46] and Pakistan [47]. *Acinetobacter* strains with *mcr-1*, *mcr-2*, and *mcr-3* genes have been detected from clinical and environmental samples in Iraq [48]. In this study, the *A. baumannii* and *A. nosocomialis* strains carried the *mcr-4.3* gene, which has already been found in *A. baumannii* strains from pig faeces in China [23], a meningitis case in Brazil [19], and human and food samples in the Czech Republic [24]. *A. nosocomialis* with *mcr-4.3* has been described sporadically so far. Currently, this species has been associated with the *mcr-4.3* gene only as NCBI database entry MG948623 (the sequence of the *mcr-4.3* gene from *A. nosocomialis* from South Africa). The common backbone of *mcr-4.3*-carrying plasmids in *Acinetobacter* spp. found in this study was described by Bitar et al. [24], where he compared *mcr-4*-positive plasmids from the Czech Republic with the ones from China [23] and Brazil [19].

The most common plasmid types associated with the *mcr-1* gene are IncX4, IncI2, and IncHI2 [49]. Of these, the IncI2 plasmid type is typical for Asia, whereas IncHI2 is typical for Europe [50,51]. Despite the Asian origin of the strains tested in this study, *mcr-1* was predominantly found on IncHI2 (n = 7), followed by two IncI2 and one each of the IncX4, IncFIB-FIC, IncFIB(K), and p0111 plasmids. A previous study on Enterobacterales strains originating from Czech retailed meat samples focused on the characterisation of plasmids carrying the *mcr-1* gene, and only the three most common plasmid types were described (IncX4, IncHI2, IncI2) [52]. Our results suggest that aquacultures and Asian countries can be a source of diversity among plasmids carrying the *mcr-1* gene.

The IncHI2 plasmids are usually hundreds of kb in size and, apart from the *mcr* gene, they carry multiple other ARGs in the multidrug-resistant (MDR) area, which varies between the plasmids while the backbone is conserved [53]. This phenomenon was also observed in this study. Interestingly, most of the IncHI2 plasmids in *E. coli* isolated from the same sample varied among each other, suggesting that the evolution of this plasmid type is very fast. Only IncHI2 plasmids from *E. coli* strains CT258 and CT262 shared a 99.98% identity in 99% coverage, being approx. 273 kb in size. The strains CT258 and CT262 originated from the same sample of frog legs but belonged to different STs (Table 1) and carried different plasmid types (Table S1).

On the other hand, the IncX4 plasmids with *mcr-1* are known to be very conserved [54], usually being approx. 33 kb in size and carrying no other resistance genes, which was also the case of IncX4 plasmid in the *E. coli* strain CT226.

The phage-like plasmid p0111 of *E. coli* strain CT229 shared a significant identity with the P1 phage (accession number AF234172). When undergoing lysogenic conversion, P1 phage does not incorporate into the chromosome but circularises as a plasmid. In this case, the transmission of the *mcr-1* gene could have been achieved via transduction, subsequently leading to phage degradation. The occurrence of *mcr-1* on phage-like plasmids has been reported before [55,56], and the *mcr-1* gene has been found within metagenomic studies of phage populations in swine feedlot wastewater [57] or chicken faeces [58]. Additionally, the CT229 phage-like plasmid showed a >99.96% identity with a >98% coverage with plasmids (accession numbers MG288678 and MF455226) from *K. pneumoniae* and *E. coli* from China, suggesting the wider spread of this phage-like plasmid.

When comparing the plasmid IncFIB(AP001918)-FIC(FII) of *E. coli* CT225 with the public database, similar plasmids were found but none of them contained the *mcr-1.1* gene (accession numbers, e.g., CP075063, AP023199, or CP055255). The *mcr-1.1* has been found to be located on IncFIB plasmid types before [59,60]; however, to our best knowledge, even though the replicon type IncFIC has been found in strains containing the *mcr-1* gene [61], the *mcr-1* gene has not been localised on the IncFIC plasmid type. Similarly, when performing BLASTn search for the IncFIB(K) plasmid of *E. coli* CT250, the most similar plasmids found belonged to the IncHI2 type (accession numbers, e.g., MG385063, MN232189, or CP019214). This suggests a rare finding of the *mcr-1* gene localised on the IncFIB-FIC and IncFIB(K) plasmid types.

Plasmid IncFIA(HI1) of *K. pneumoniae* CT251 showed a 99.61% identity in 73% coverage with MK262711.1, a larger plasmid p18-29mcr-8.2 (approx. 91 kb in size) of *K. pneumoniae* KP18-29 from a human urine sample from China carrying the *mcr-8.2* gene. The transposon Tn*6330* of plasmid CT251 was not present in p18-29mcr-8.2. The surroundings of the *mcr-8.2* gene were found to be relatively conserved [62], and plasmid CT251 shared some previously described features: *mcr-8.2* was flanked by IS*903* and IS*Kpn26* and the genes *dgkA* and *copR* were found upstream of the *mcr-8.2* gene. In *K. pneumoniae* strain CT251, Tn*6330* was located between the *mcr-8.2* gene and IS*903*. These findings suggest that *Tn6330* with the *mcr-1.1* gene was incorporated into the *mcr-8.2*-bearing plasmid in strain CT251.

The mobilisation of ARGs is often achieved using insertion sequences. In the case of the *mcr-1* gene, IS*Apl1* has been found co-localised with *mcr-1* forming a transposon Tn*6330* when localised upstream and downstream of the gene [63]. In this study, different types of genetic arrangements around the *mcr-1* gene were found (Figure 2), which is consistent with the previously described surroundings of the *mcr-1* gene [63,64] and show the possibility of its transfer between plasmids and/or chromosomes.

The *mcr-3.5*-carrying plasmid IncFII(pCoo) in *E. coli* CT262 from frog legs imported to the Czech Republic from Vietnam was 99.9% identical in 87% coverage with AP018353—an *mcr-3.2* gene carrying IncFII plasmid from pork meat from Vietnam [65], suggesting a possible dissemination of these plasmids carrying *mcr-3* gene in the country. Another study in China [66] characterised *E. coli* strains positive for both *mcr-1* and *mcr-3* and localised these genes on different plasmids or chromosomes. The plasmid pCP55-IncFII with *mcr-3.5* shared a 95.85% identity in a 60% coverage with the *mcr-3.5* carrying plasmid of strain CT262 from this study.

The occurrence of *mcr* genes in the chromosome is not observed very often [64] but can represent a threat of stabilising the heritage of *mcr-1* [67]. The chromosomal carriage of the *mcr-1* gene has been detected in 36.8% of *mcr-1*-positive *E. coli* strains isolated from healthy residents in Vietnam [68] and found in two *E. coli* strains isolated in a medical setting in Vietnam [69]. In this study, the *mcr-1* gene was found on the chromosome in two *E. coli* strains, CT227 and CT230, originating from pangasius and crab meat from Vietnam, respectively. As in the case of the phage-like plasmid p0111 of *E. coli* strain CT229, the strain CT227 with chromosomally located *mcr-1* could have acquired the *mcr-1* gene by lysogeny of phage. Similarly, it has been observed by Shen et al. that the most common phage-like region around the *mcr-1* gene contains an incomplete phage Vibrio 12B8 (NC_021073), as found by Phaster [67]. In contrast to the tested *E. coli* strains with the *mcr-1* gene on plasmids, strain CT227 carried only the IncY-type plasmid, along with some small replicons of a few kb in size. The strain CT230 did not carry any plasmids at all (Table S1). In a recent study on Czech travellers and expatriates living in the Czech Republic, one *E. coli* strain with *mcr-1* in the chromosome was found and the strain carried only one additional plasmid with no ARGs [70]. Even though the occurrence of *mcr* genes in the chromosome is quite rare, it could represent a heritable repository and emerge again if new selective pressure appears.

## 4. Materials and Methods

### 4.1. Bacterial Isolates Collection with Colistin Resistance

In this study, seventeen colistin-resistant bacterial isolates originating from aquaculture products imported from Vietnam were analysed. The isolates were obtained from 53 retailed originally packed samples in the Czech Republic throughout the year 2019; out of these, four were positive for *mcr*-carrying bacteria (unpublished data). The *mcr*-positive isolates were detected in aquaculture products originating from Vietnam but from different producers. The samples of pangasius and crab meat originated from aquaculture products caught in freshwaters, whereas the samples of frog legs came from farmed frogs.

The minimum inhibitory concentration (MIC) of colistin was determined by the microdilution method (Erba Lachema, Brno, Czech Republic) and evaluated according to EUCAST (European Committee on Antimicrobial Susceptibility Testing, 2019, https://www.eucast.org/clinical_breakpoints/, accessed on 7 April 2020).

### 4.2. Genomic DNA Extraction, Whole-Genome Sequencing (WGS), and Genome Assembly

For short-read whole-genome sequencing, genomic DNA was extracted using the DNeasy Blood and Tissue kit according to the manufacturer’s instructions (Qiagen, Hilden, Germany). The preparation of DNA libraries and sequencing on the Illumina platform were carried out by LGC Genomics GmbH group (NextSeq, 2 × 150 bp).

To determine the localisation of *mcr* genes, Oxford Nanopore Technologies (ONT, Oxford, UK) long-read sequencing was applied. The genomic DNA was extracted using the MagAttract HMW DNA Kit (Qiagen, Hilden, Germany). MinION libraries were prepared with a Ligation Sequencing Kit, #SQK-LSK109, (ONT, Oxford, UK) and sequenced in a #FLO-MIN106 R9.4 flow cell. Fast5 read files were base called and converted to fastq format using the software Guppy v 3.0.3+7e7b7d0 (ONT). The de novo hybrid assembly of long (ONT) and short (Illumina) reads was conducted using Unicycler v0.4.7 [71]. The contigs were checked for circularisation and size.

### 4.3. Multilocus Sequence Typing (MLST)

*E. coli* sequence type was determined by the Achtman MLST scheme (www.enterobase.warwick.ac.uk/species/e.coli, accessed on 17 June 2020), whereas the Pasteur MLST scheme was used for the *Klebsiella pneumoniae* isolate (https://bigsdb.pasteur.fr/klebsiella/klebsiella.html, accessed on 17 June 2020) and *Acinetobacter* spp. isolates (https://pubmlst.org/abaumannii/, accessed on 17 June 2020).

### 4.4. Genetic Analysis of Plasmids and Antibiotic Resistance Genes

Plasmid types and resistance genes in Enterobacterales isolates were evaluated using PlasmidFinder [39] and ResFinder [40,72], available at https://cge.cbs.dtu.dk/services/ (accessed on 22 September 2020). For *Acinetobacter* spp. isolates, the ARGs were analysed by CARD [73] (https://card.mcmaster.ca/analyze/rgi, accessed on 22 September 2020). The annotation of genes was carried out using the Prokka v1.13.7 software [74] and RAST software [75] (https://rast.theseed.org/FIG/rast.cgi, accessed on 9 December 2020). The plasmids of identical type in this study were compared between each other using BRIG [76] v0.95 (Blast Ring Image Generator, http://brig.sourceforge.net/, accessed on 10 December 2020). BLASTn (https://blast.ncbi.nlm.nih.gov/Blast.cgi, accessed on 10 December 2020), with default parameters, was used on unique *mcr*-carrying plasmid sequences from this study to search for similar plasmids available in the NCBI database. PHASTER [38] (PHAge Search Tool Enhanced Release, https://phaster.ca/, accessed on 14 December 2020) was used to identify and annotate prophage sequences possibly surrounding the *mcr* genes.

## 5. Conclusions

The *mcr-1.1* gene was found located on *mcr-1*-associated plasmid types IncHI2, IncI2, and IncX4, as well as on the rarely described plasmid types IncFIB-FIC and IncFIB(K), phage-like plasmid p0111, and on the chromosome of *E. coli* from retailed aquaculture products imported to the Czech Republic from Vietnam. One *E. coli* strain carried the *mcr-3.5* gene on IncFII(pCoo) plasmid in addition to the *mcr-1.1* gene located on IncHI2 plasmid. The *mcr-4.3* gene was found on similar plasmids of *A. baumannii* and *A. nosocomialis* strains, pointing to the possible interspecies transfer of plasmids carrying the *mcr-4.3* gene. *K. pneumoniae* was found to carry the *mcr-1.1* and *mcr-8.2* genes on IncFIA(HI1) plasmid. This study highlights the risks involved in the spreading of bacteria resistant to colistin, being a last-resort antibiotic, as well as having other resistances, such as genes encoding resistance to beta-lactams or fluoroquinolones from aquaculture sources of Asian origin.

## Figures and Tables

**Table 1:** Summary table of the tested bacterial strains with *mcr*-mediated colistin resistance originating from aquaculture products.

Strain ID	Source	Species	Colistin MIC (mg/L)	MLST	*mcr* Gene	*mcr* Gene Localisation (Plasmid Type/ Chromosome)
CT225	pangasius	*E. coli*	4	ST155	*mcr-1.1*	IncFIB(AP001918)-FIC(FII)
CT226	pangasius	*E. coli*	>16	ST2253	*mcr-1.1*	IncX4, IncI2
CT227	pangasius	*E. coli*	4	ST206	*mcr-1.1*	chromosome
CT228	pangasius	*E. coli*	8	ST156	*mcr-1.1*	IncI2
CT229	crab	*E. coli*	4	ST1011	*mcr-1.1*	p0111
CT230	crab	*E. coli*	8	ST6745	*mcr-1.1*	chromosome
CT248	frog legs	*E. coli*	4	ST4481	*mcr-1.1*	IncHI2
CT249	frog legs	*E. coli*	4	ST48	*mcr-1.1*	IncHI2-N
CT250	frog legs	*E. coli*	4	ST2179	*mcr-1.1*	IncFIB(K)
CT258	frog legs	*E. coli*	4	ST48	*mcr-1.1*	IncHI2
CT259	frog legs	*E. coli*	8	ST8680	*mcr-1.1*	IncHI2-N
CT260	frog legs	*E. coli*	4	ST48	*mcr-1.1*	IncHI2
CT262	frog legs	*E. coli*	4	ST609	*mcr-1.1*, *mcr-3.5*	*mcr-1*/IncHI2, *mcr-3*/IncFII(pCoo)
CT267	frog legs	*E. coli*	4	ST48	*mcr-1.1*	IncHI2
CT251	frog legs	*K. pneumoniae*	>16	ST11	*mcr-1.1* + *mcr-8.2*	IncFIA(HI1)
CT237	pangasius	*A. nosocomialis*	>16	ST279	*mcr-4.3*	untypeable plasmid
CT263	frog legs	*A. baumannii*	>16	ST490	*mcr-4.3*	untypeable plasmid

## Data Availability

The sequencing data after hybrid assembly are available at Mendeley data, doi:10.17632/gj4pg2rbtp.1.

## Supplementary Materials

The following are available online at https://www.mdpi.com/article/10.3390/antibiotics10070838/s1: Table S1: Detailed localisation of antimicrobial resistance genes in tested strains.
